# Abnormal Brain Default-Mode Network Functional Connectivity in Drug Addicts

**DOI:** 10.1371/journal.pone.0016560

**Published:** 2011-01-26

**Authors:** Ning Ma, Ying Liu, Xian-Ming Fu, Nan Li, Chang-Xin Wang, Hao Zhang, Ruo-Bing Qian, Hu-Sheng Xu, Xiaoping Hu, Da-Ren Zhang

**Affiliations:** 1 CAS Key Laboratory of Brain Function and Disease, and School of Life Sciences, University of Science and Technology of China, Hefei, China; 2 Department of MRI at Provincial Hospital Affiliated to Anhui Medical University, Hefei, China; 3 Department of Neurosurgery at Provincial Hospital Affiliated to Anhui Medical University and Anhui Provincial Institute of Stereotactic Neurosurgery, Hefei, China; 4 Anhui Detoxification and Rehabilitation Center, Hefei, China; 5 The Wallace H. Coulter Department of Biomedical Engineering, Georgia Institute of Technology and Emory University, Atlanta, Georgia, United States of America; The University of Melbourne, Australia

## Abstract

**Background:**

The default mode network (DMN) is a set of brain regions that exhibit synchronized low frequency oscillations at resting-state, and is believed to be relevant to attention and self-monitoring. As the anterior cingulate cortex and hippocampus are impaired in drug addiction and meanwhile are parts of the DMN, the present study examined addiction-related alteration of functional connectivity of the DMN.

**Methodology:**

Resting-state functional magnetic resonance imaging data of chronic heroin users (14 males, age: 30.1±5.3 years, range from 22 to 39 years) and non-addicted controls (13 males, age: 29.8±7.2 years, range from 20 to 39 years) were investigated with independent component analysis to address their functional connectivity of the DMN.

**Principal Findings:**

Compared with controls, heroin users showed increased functional connectivity in right hippocampus and decreased functional connectivity in right dorsal anterior cingulate cortex and left caudate in the DMN.

**Conclusions:**

These findings suggest drug addicts' abnormal functional organization of the DMN, and are discussed as addiction-related abnormally increased memory processing but diminished cognitive control related to attention and self-monitoring, which may underlie the hypersensitivity toward drug related cues but weakened strength of cognitive control in the state of addiction.

## Introduction

Addictive drug use is often related to abnormal functional organization in the users' brain, which leads to habitually hypersensitivity to drug-related cues and ensures their compulsive patterns of drug-seeking behavior [1]. The brain circuits of memory and learning, including the hippocampus, and cognitive control, involving the dorsal anterior cingulate cortex, are believed to be impaired in addiction. As a result, under addiction, saliency value of drug-related cues are enhanced, while inhibitory controls are weakened, setting up the stage for an unrestrained cycle which leads to compulsive drug-seeking without regard to its negative consequences [2], [3], [4], [5], [6], [7].

The default mode network (DMN) is a set of brain regions that exhibit robust low frequency oscillations coherent during resting-state and typically deactivate during performance of cognitive tasks [8], [9]. Brain regions identified as parts of the DMN include the cingulate cortex, hippocampus and medial prefrontal and inferior parietal cortices [8], [10]. As the hippocampus and anterior cingulate cortex are impaired in addiction [2], [3], [4], [5], [6], [7], we hypothesized that the DMN would exhibit abnormal functional organization in drug addicts, especially in brain regions which have been shown to have altered function in previous studies of addiction [11], [12], [13]. However, to date, the DMN is poorly characterized in drug addicts.

Resting-state functional connectivity, assessed by the correlation of spontaneous fluctuations of blood oxygen level-dependent (BOLD) signals in different regions of the “resting” brain, is believed to provide a measure of the brain's functional organization [8]. The DMN can be readily identified by using this approach with the algorithm of independent component analysis (ICA, although recent evidence suggests that the DMN is composed of a number of sub-networks [14]) [10]. Therefore, the present study investigated possible addiction related alteration of functional connectivity of the DMN with functional magnetic resonance imaging (fMRI) data acquired during resting state from chronic heroin users and non-addicted controls using ICA. Compared with controls, we found increased functional connectivity in hippocampus and decreased functional connectivity in dorsal anterior cingulate cortex and caudate in the DMN in heroin users.

## Materials and Methods

### Participants

The fMRI dataset we examined in the present study is identical with that analyzed in our previous report [15]. Twenty seven right-handed male volunteers, including 14 chronic heroin users [HU, heroin using (from the time of their initial heroin use until the time of scanning) for 7.11±2.82 years, range from 2 to 10 years] and 13 non-addicted controls (CN), participated in the present study. All HU were recruited from Anhui Detoxification and Rehabilitation Center (Hefei, Anhui province, China), sought medical help on their own initiative and had a DSM-IV diagnosis of heroin dependence or abuse and urine tests positive for heroin before enrolling in the treatment program. According to an interview conducted by a clinical psychologist, all of the patients had never used any other types of illicit drugs, were free of illnesses that required hospitalization or regular monitoring and were deemed to be stable and able to participate in the experiment. Before the fMRI scanning, except 2 of HU who were current heroin users and not under any treatment, the HU were under a methadone treatment and had no illicit drug use during the treatment as confirmed by their care takers. Among the 12 HU under the treatment, 9 were in the detoxification phase (entering the program within one week before the scanning, mean  = 2.1 days, SD  = 1.8), while the remaining 3 were in a relatively stable maintenance phase (one had been in the program for 2 months and the other two for 6 months). All methadone-treated participants were under daily methadone administrations and their last methadone uses were at least 12 hours before the scanning.

The control participants were recruited through advertisements and compensated for their time, and none of them reported a history of head injury, psychiatric disorders or substance dependence (other than cigarette smoking). Only male participants were selected as gender effect was not a focus of our present study. Both cohorts of HU and CN were current tobacco users. The HU and CN were matched in age (HU, 30.1±5.3 years, range from 22 to 39 years; CN, 29.8±7.2 years, range from 20 to 39 years; *t*(25) = 0.093, ns) and years of education (HU, 9.71±2.7, range from 5 to 14; CN, 10.8±1.6, range from 8 to 13; *t*(25) = −1.296, ns). This study was approved by the review board of University of Science & Technology of China. After complete description of the study to the participants, written informed consent was obtained from them for their involvement in this study in accordance with the review board of University of Science & Technology of China.

### Scanning and image preprocessing

Please see Supplementary Materials S1 for the details of scanning and image preprocessing. Briefly, resting-state fMRI data were acquired (eye closed) with a T2*-weighted echo-planar imaging sequence (TE  = 30 ms, TR  = 2 s, FOV  = 24 cm, Matrix = 64×64) with 22 axial slices (slice gap  = 0.4 mm, voxel size: 3.75×3.75×4 mm^3^) in one run of six minutes on a 3T Siemens Magnetom Trio scanner (Siemens Medical Solutions, Erlangen, Germany). The preprocessing of fMRI data was similar to that used in our previous report [15]. For each participant, with the software of Analysis of Functional Neuroimages (AFNI) [16], the raw data were corrected for temporal shifts between slices, corrected for head motion, spatially smoothed and temporally normalized. Motion data were regressed out of the time series and band pass temporal filtering (0.01 Hz to 0.08 Hz) was performed on the residual signals [17], [18]. These preprocessed time series were used in the subsequent functional connectivity analysis.

### Functional Connectivity Analysis

To ascertain functional connectivity within the DMN, the preprocessed time series were analyzed with the algorithm of ICA using fMRIb software library's (FSL) implementation of MELODIC (www.fmrib.ox.ac.uk/fsl/melodic2/index.html). As yet, there is no consensus on how to choose the optimal number of components, although methods to do so are in development [19]. In the present study, although we set the output of components into 25 (approximately 1/7 the number of time points in the respective scans) in the software, the ICA software could not converge on 25 components in all of 27 participants and the number of components generated ranged from 20 to 28 (HU, from 20 to 28, Mean  = 23.5; CN, from 20 to 26, Mean  = 23.3, independent between-group *t*-test, *t*(25) = 0.224, ns). To select the DMN component in each participant, the automated process introduced by Greicius' group [10], [20], [21], [22] was used. First, by visually inspection, the component whose *z*-score map was most similar to the map of the DMN was chosen. These maps were transformed to Talairach space [23] (re-sampled voxel size: 3×3×3 mm^3^) according to the spatial transformation between the anatomic data and the Talairach space, and, using AFNI, a voxel-wise one sample *t*-test was performed across these Talairach-transformed maps of all 27 participants to generate a “template of the DMN” containing voxels whose *z*-scores were significantly larger than zero in the *t-*test (determined by combining individual voxel threshold of *p*<0.005 with a spatial cluster size of 56 voxels which yielded a false-positive level of 0.05 for whole brain voxels according to a Monte Carlo simulation conducted with AFNI). Second, this template was used to select the “best-fit” component of the DMN in each participant. To do so, we used a “linear template-matching procedure” that involves taking the average *z-*score of voxels falling within the template minus the average *z*-score of voxels outside the template and selecting the component in which this difference (the goodness-of-fit) was the greatest [10], [20], [21], [22]. This procedure was done in the image space of each participant, and “template of the DMN” was transformed into the image space of each participant before this step. In order to test how well this approach worked in selecting a uniquely representative default-mode component and to be certain that the approach did not differ between groups, we compared the mean goodness-of-fit scores for the best-fit component and the second best-fit component, respectively, within and between groups using paired and two-sample *t*-tests. “Best-fit” component images of each participant were shown in Supplementary Materials S1. These images indicated that the application of ‘linear template-matching procedure’ in our data was an appropriate method for extracting the DMN map for each participant, similar to the results of several previous studies [10], [20], [21].

### Group Analysis

All group analyses were performed on the participants' “best-fit” component images. First, these maps were transformed to Talairach space (re-sampled voxel size: 3×3×3 mm^3^) according to the spatial transformation between the anatomic data and the Talairach space. Second, using AFNI, voxel-wise one-sample *t*-tests and a two-sample *t*-test were performed to compare the DMN within and between the two groups. For the one-sample *t*-tests, the individual voxel-wise *z*-score maps (assigned by ICA) of the best-fit component for each participant in the group (HU or CN, respectively) were entered into group-level one-sample *t*-test against zero. Clusters with *z*-scores significantly larger than zero were determined by combining individual voxel threshold of *p*<0.005 with a spatial cluster size of 64 or 71 voxels (HU or CN) which yielded a false-positive level of 0.05 for the entire volume according to Monte Carlo simulations conducted with AFNI. For the two-sample *t*-test, the individual voxel-wise *z*-score maps of the best-fit component for each participant in the two groups were entered into group-level two-sample *t*-test compared between groups. Since we only focused on the between-groups difference within the DMN, the two-sample *t*-test was restricted (masked) to the voxels within the DMN as defined by a logical ‘OR’ between the two group-maps of the DMN resulting from the one-sample *t*-tests described above [20], [21]. Clusters with *z*-scores significantly differed between two groups were determined by combining individual voxel threshold of *p*<0.005 with a spatial cluster size of 12 voxels which yielded a false-positive level of 0.05 for voxels in the mask according to a Monte Carlo simulation conducted with AFNI.

## Results

### Goodness-of-Fit Scores

**Figure 1:**
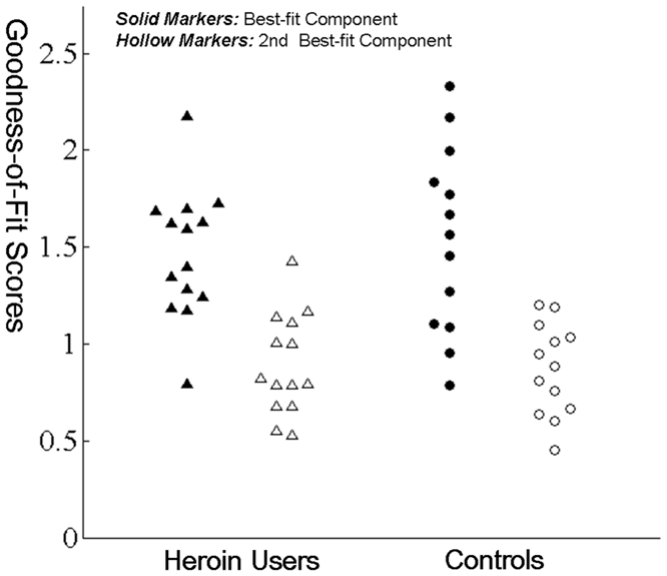
Scatter Plots of the Goodness-of-fit Scores. Triangles  =  Heroin users, Circles  =  Controls; Solid markers: Goodness-of-fit Scores of best–fit component, Hollow markers: Goodness-of-fit Scores of second best–fit component.

Across the 27 participants, the goodness-of-fit score was 1.60±0.43 (Mean ± SD) for the best-fit component which was significantly higher than that for the second best-fit component (0.93±0.26, *t*(26) = 6.147, *p*<0.001). The two groups did not differ in their mean goodness-of-fit score for the best-fit component (HU, 1.63±0.51, CN, 1.57±0.36, *t*(25) = 0.391, ns) or the second best-fit component (HU, 0.91±0.24, CN, 0.95±0.28, *t*(25) = −0.404, ns). These results suggest that the automated selection procedure was effective in selecting a unique component in each participant that corresponded to the DMN, and that the selection procedure worked equally well between the two groups [20]. The scatter plots of the goodness-of-fit scores were shown in Figure 1.

### Default-mode network

**Figure 2:**
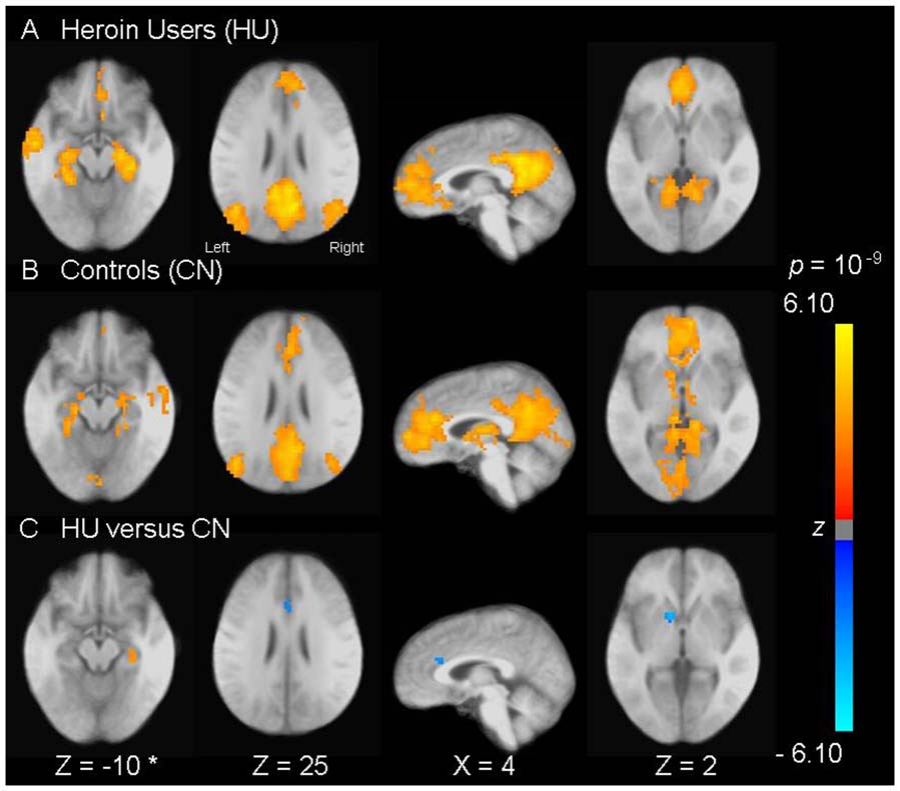
Altered Functional Connectivity of the Default-Mode Network in Heroin Users. Images of group default-mode functional connectivity in heroin users (HU; A) and in non-addicted controls (CN; B). The contrast map in panel C demonstrates clusters (see Table 1) where resting-state functional connectivity in the DMN was altered in HU versus CN. Clusters above thresholds (*p*<0.05, corrected) in *t*-tests were lightened with *z*-scores converted from *t*-scores of these tests. The *z*-score bar is shown right side of the figures. The left side of these axial images corresponds to the left side of the brain. * Coordinates in Talairach space (Z, Inferior to Superior, X, Left to Right). For display purposes, a single, averaged, skull striped, stereotaxic transformed image was derived from 27 three-dimensional structural scans taken from all participants of both groups and statistical maps were superimposed on this structural image.

Within the DMN, in contrast to controls, heroin users showed significantly more connectivity from right hippocampus, but significantly reduced connectivity from right dorsal anterior cingulate cortex (ACC) and left caudate to the DMN (Table 1, Figure 2).

**Table 1:** Significant Clusters in the Voxel-Wise Two-Sample *t*-test Comparing Functional Connectivity of the Default-Mode Network in Heroin Users (HU) versus Controls (CN).

Cluster	Cluster	Primary	Within-group *z*-scores b			
Anatomical Locations	Size	Peak	HU (n = 14)		CN (n = 13)	
	(mm^3^)	Location a	Mean	SD	Mean	SD
HU > CN						
right hippocampus	324	31.5, −25.5, −9.5	1.24	0.44	0.21	0.64
HU < CN						
right dorsal ACC (BA 24)	405	4.5, 25.5, 23.5	0.10	0.77	1.61	1.08
left caudate	351	−10.5, 10.5, 2.5	−0.28	0.55	0.88	0.55

a. Coordinates in Talairach space (Left to Right, Posterior to Anterior, Inferior to Superior).

b. Within-group mean-values and standard deviations of *z*-scores for each cluster; for each participant, the *z*-score for each cluster was calculated by averaging *z*-scores of all voxels in that cluster. BA  =  Brodmann's Area, ACC  =  anterior cingulate cortex.

## Discussion

With resting-state fMRI data acquired from chronic heroin users and non-addicted controls, addiction related alteration of functional connectivity of the DMN is demonstrated. The algorithm of independent component analysis and voxel-based measure used in the present study reflects the degree to which a particular voxel's time series is correlated with the overall time series of the entire DMN. Thus, following the reasoning in Greicius's study [20], we interpret our findings as showing that the DMN functional connectivity in drug addicts is disproportionately driven by the clusters which exhibited between-group differences.

As we hypothesized, compared with controls, brain regions previously identified to be impaired in drug addiction showed abnormal functional connectivity to the DMN in drug addicts in the present study. These findings could be interpreted in light of the addiction-related disruption of brain function and the putative functional roles of the DMN. The DMN is believed to be relevant to attention, self-monitoring and introspective thoughts and represent a “sentinel” role of the resting state that awakes brain broadly to evaluate information from external and internal environment [9]. A recent study using task-based meta-analyses [24] suggest that the DMN is similar to those identifiable with many task paradigms. Although there is recent evidence indicating that there is no one to one correspondence between functional and structural connectivity [25], it is suggested that the functional connectivity in the DMN may reflect a structural connectivity in the network [22]. Altered functional connectivity of the DMN has been reported in a number of studies of neuropsychiatric disorders including schizophrenia, attention-deficit hyperactivity disorder, depression, anxiety, autism spectrum disorder, and Alzheimer's disease [10], [20], [26], [27], [28], [29] and is typically characterized by dysfunction of introspective mental processes which may be potential sources of interference during goal-directed activity (attentional orientation) [30].

Hippocampus is the main brain structure involved in learning and memory, and thought to primarily contribute to the acquisition, consolidation and expression of learning of the drug related cues that drives relapse to drug-seeking behaviors [13], [31]. The hippocampus is also a prominent node within the DMN [10]. In the present study, the increased hippocampal functional connectivity within the DMN in drug addicts may suggest abnormally enhanced memory processing, which may disturb the addicts' attentional orientation and self-monitoring during resting state. This finding may underlie the addicts' hypersensitivity on drug-related cues driven by abnormal memory processing [13], [31].

Dorsal anterior cingulate cortex (dACC) has long been recognized as an important structure in cognitive control and error monitoring [32]. In drug addicts, dysfunction in dACC was found to be linked to their compromised ability in inhibitory control [11], [33]. In the present study, in drug addicts, the dACC showed decreased contributions of functional connectivity in the DMN. This result suggests that, as a region impaired in addiction, dACC may be less recruited by the functions of the DMN such as attentional orientation and self-monitoring in drug addicts during resting state, which may underlie addiction-related weakened cognitive control.

We also found decreased functional connectivity in the striatal region, caudate, in the DMN in heroin users. The function of striatal regions is believed to be impaired in drug addiction [34], [35], [36]. A recent study proposed that striatal dopamine circuits, particularly the caudate, may provide a mechanism for the active suppression of the DMN under conditions that require increased processing of external stimuli relative to internal, self-directed processing [37]. Previous studies have demonstrated that the activity and functional connectivity within the DMN is reduced during task performance and this reduction typically occurs in association with increased activation in task-relevant regions [38], [39]. Failure to suppress the DMN during behavioral tasks was found to be related to impaired performance [40], [41]. Thus, we conjecture that our finding of drug addicts' decreased functional connectivity in the caudate might underlie their dysfunction in suppressing the DMN during cognitive tasks. This notion may give a possible explanation about addiction-related impaired attentional orientation and cognitive control toward goal-directed activities [42]. However, as we had no external measures of the addicts' cognitive performance in the present study and the functional role of caudate in the DMN is still to be clarified, further studies are needed to provide a more definitive interpretation of our present finding.

The fMRI dataset we examined in the present study was identical with those we analyzed in our previous study [15], however, these two studies were aimed at different questions. In our previous study, we indentified addicts' abnormal functional connectivity among the brain regions including nucleus accumbens, amygdale, orbital frontal cortex and ACC. In the present study, however, we detected addiction-related alteration of brain default-mode network functional connectivity. Altered functional connectivity of the dorsal ACC was indentified in both our previous and present studies. This region exhibited decreased connectivity with the ventral ACC, the dorsolateral prefrontal cortex [15] and the DMN (present study) in drug addicts. These results suggested a critical role of the dorsal ACC, particular its functional connectivity, in drug addiction, and further studies to explore the functional and clinical significance of our findings are warranted.

As in our previous study [15], additional investigations involving larger sample size of and more diverse participants are needed for generalizing the results of the present study, examining the relationship between behavioral and imaging data, and clarifying the effects of different treatment stages of addiction on functional organization of the DMN. In particular, the present study did not dissociate the effects of methadone treatment from those of heroin addiction, so further studies in this regard is needed; such a study may be of potentially clinical significance (please see Supplementary Materials S1 for detailed discussion about the effect of methadone). Meanwhile, more advanced data acquiring and analysis methods (such as ‘dual regression approach’ for extracting the DMN [43], [44]) and combination with diffusion tensor imaging [22] may be used in future studies.

Taken together, the abnormality of functional connectivity of the DMN we found in drug addicts suggest drug addicts' altered functional organization of the DMN, which may be implicated in addiction-related increased memory processing and diminished cognitive control related to attention and self-monitoring. These notions may underlie the addicts' hypersensitivity to drug related cues and weakened strength of cognitive control [2], [3], [4], [5], [6], [7].

## Supporting Information

Supplementary Materials S1 (DOC)
