# Supplementary material for: Abnormal Brain Default-Mode Network Functional Connectivity in Drug Addicts
Source: PLoS One. 2011 Jan 26;6(1):e16560. doi: 10.1371/journal.pone.0016560 (PMC3027699; doi:10.1371/journal.pone.0016560)
Supplement: Supplementary Materials S1 — (DOC) [file pone.0016560.s001.doc]

**Supplementary Material**

**Scanning and image preprocessing**

All imaging data were obtained on the 3T Siemens Magnetom Trio scanner (Siemens Medical Solutions, Erlangen, Germany) in the Anhui Provincial Hospital (Hefei, Anhui, China). A circularly polarized head coil was used, with foam padding to restrict head motion. Functional images were acquired with a T2*-weighted echo-planar imaging sequence (TE = 30ms, TR = 2s, FOV = 24cm, Matrix=64×64) with 22 axial slices (slice gap = 0.4 mm, voxel size: 3.75×3.75×4 mm^3^), covering the parietal lobe, the occipital lobe and a large portion of the frontal lobe and the temporal lobe (the slices were approximately along the AC-PC line and covered about -30 to 60 in the Inferior-Superior direction). Resting-state fMRI data were acquired with one run of six minutes (180 images per slice). Corresponding high-resolution T1-weighted spin-echo (for anatomical overlay) images and three-dimensional gradient-echo (for stereotaxic transformation) images were also collected. Before entering the scanner, all participants were told to close their eyes, remain still and relaxed, and stay awake during the scanning. After the resting-state scanning, several functional activation runs were acquired with cognitive tasks (data to appear elsewhere). All participants could response to these tasks immediately after the resting-state scanning suggesting they may not asleep during the resting state. Before and after the scanning, according to the reports of a clinical psychologist, all the heroin users were ensured to be at a stable state and not intoxicated during the scanning.

The imaging data were mainly processed with Analysis of Functional Neuroimages (AFNI) [1]. For each participant, the first four time points were discarded to account for the approach to steady state in the BOLD signal. The raw data were corrected for temporal shifts between slices, corrected for head motion, spatially smoothed with a Gaussian kernel (full width at half maximum = 4 mm) and temporally normalized (for each voxel, the signal of each image was divided by the temporally averaged signal). We totally scanned 17 HU and 16 CN participants and discarded the data of participants whose head moved more than 1.0 mm in any dimension through the resting-state run. Data of 14 HU and 13 CN participants met the movement criterion and appeared in the present study. To further reduce the effect of motion and obtain low frequency fluctuation, we regressed the motion data out of the time series and then preformed band pass temporal filtering (0.01Hz to 0.08 Hz) on the residual signals [2,3]. In the present study, during the preprocessing of fMRI data, we did not address the potential noise due to low-frequency fluctuations from possible thermal noise, scanner instability, or alias of physiologic signals. Because the approach of independent component analysis (ICA), which we used to measure the default-mode network, was proven to be able to detect the components of these noise which are distinct from components that represent resting-state neural networks [4,5].

**Group and individual “best-fit” component images, HU = heroin users, CN = controls**

These images indicated that the application of ‘linear template-matching procedure’ in our data was an appropriate method for extracting the default mode network for each participant, similar to the results of several previous studies [6,7,8].

**
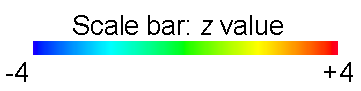
**

**
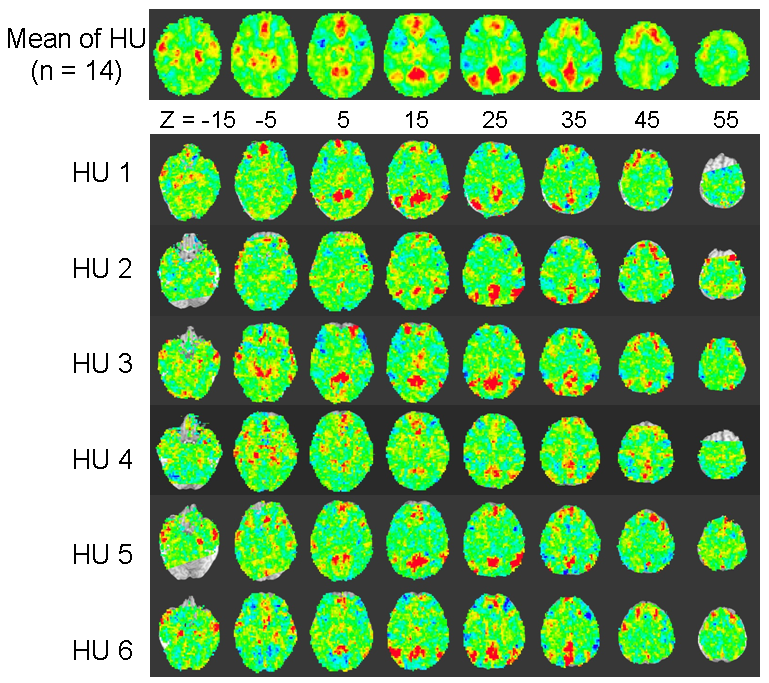
**

**
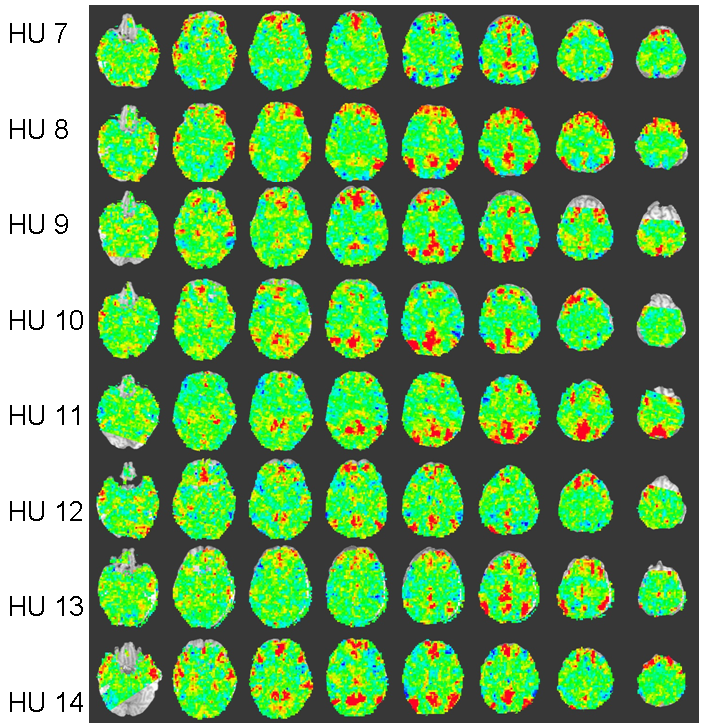
**

**
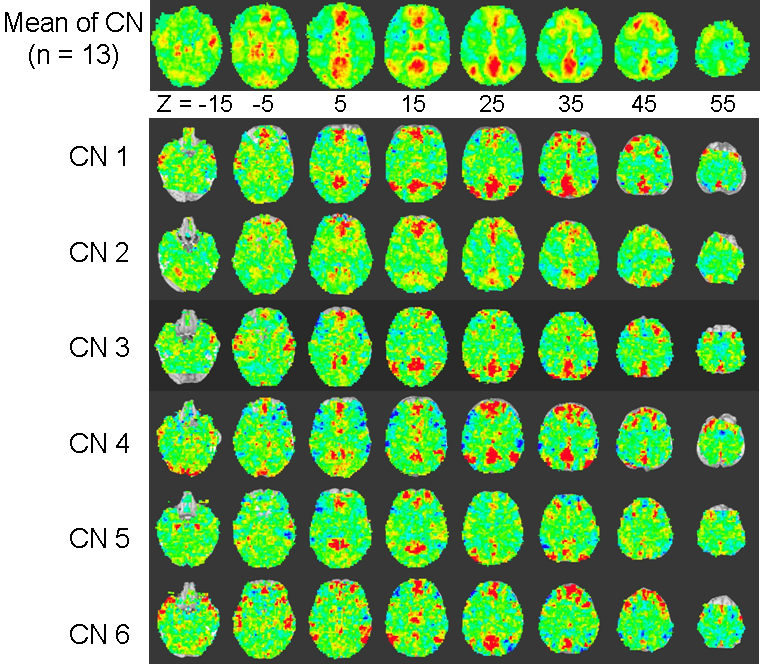
**

**
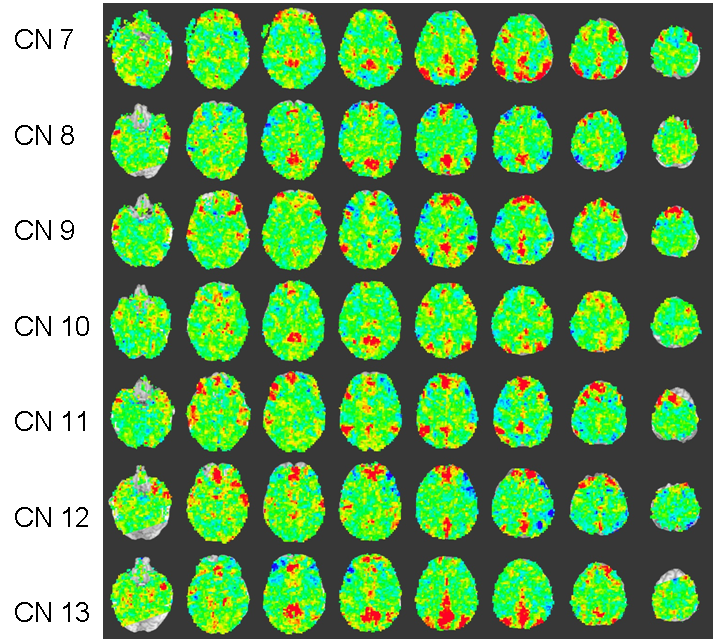
**

**Discussion about the effect of methadone**

As most heroin users in the present study were under methadone treatment, what effects of methadone would have on the functional connectivity in the DMN is an interesting question*.* Known as a drug that prolongs opioid dependence, methadone has similar effects as addictive drugs to some extent [9]. For example, studies have found that methadone can prime heroin cue response and craving [10] and enhance brain response to drug cues [11], which are similar to those in untreated heroin dependence [12]. Thus, we speculate that the alteration of the DMN related to methadone might be in a same pattern as that related to heroin addiction. However, as the effect of methadone was not sufficiently controlled in the present study, its effect on functional connectivity of the DMN needs to be clarified in further studies. Nonetheless, as heroin users under methadone treatment are thought to be still at states of addiction [9], our findings of alteration in functional connectivity in the DMN in drug addicts may be mainly related to their opioid addiction.

**References in the Supplementary Material:**

1. Cox RW (1996) AFNI: software for analysis and visualization of functional magnetic resonance neuroimages. Comput Biomed Res 29: 162-173.

2. Auer DP (2008) Spontaneous low-frequency blood oxygenation level-dependent fluctuations and functional connectivity analysis of the 'resting' brain. Magn Reson Imaging 26: 1055-1064.

3. Birn RM, Diamond JB, Smith MA, Bandettini PA (2006) Separating respiratory-variation-related fluctuations from neuronal-activity-related fluctuations in fMRI. Neuroimage 31: 1536-1548.

4. Beckmann CF, DeLuca M, Devlin JT, Smith SM (2005) Investigations into resting-state connectivity using independent component analysis. Philos Trans R Soc Lond B Biol Sci 360: 1001-1013.

5. De Luca M, Beckmann CF, De Stefano N, Matthews PM, Smith SM (2006) fMRI resting state networks define distinct modes of long-distance interactions in the human brain. Neuroimage 29: 1359-1367.

6. Greicius MD, Flores BH, Menon V, Glover GH, Solvason HB, et al. (2007) Resting-state functional connectivity in major depression: abnormally increased contributions from subgenual cingulate cortex and thalamus. Biol Psychiatry 62: 429-437.

7. Greicius MD, Kiviniemi V, Tervonen O, Vainionpaa V, Alahuhta S, et al. (2008) Persistent default-mode network connectivity during light sedation. Hum Brain Mapp 29: 839-847.

8. Greicius MD, Srivastava G, Reiss AL, Menon V (2004) Default-mode network activity distinguishes Alzheimer's disease from healthy aging: evidence from functional MRI. Proc Natl Acad Sci U S A 101: 4637-4642.

9. Connock M, Juarez-Garcia A, Jowett S, Frew E, Liu Z, et al. (2007) Methadone and buprenorphine for the management of opioid dependence: a systematic review and economic evaluation. Health Technol Assess 11: 1-171, iii-iv.

10. Curran HV, Bolton J, Wanigaratne S, Smyth C (1999) Additional methadone increases craving for heroin: a double-blind, placebo-controlled study of chronic opiate users receiving methadone substitution treatment. Addiction 94: 665-674.

11. Langleben DD, Ruparel K, Elman I, Busch-Winokur S, Pratiwadi R, et al. (2008) Acute effect of methadone maintenance dose on brain FMRI response to heroin-related cues. Am J Psychiatry 165: 390-394.

12. Daglish MR, Weinstein A, Malizia AL, Wilson S, Melichar JK, et al. (2003) Functional connectivity analysis of the neural circuits of opiate craving: "more" rather than "different"? Neuroimage 20: 1964-1970.
